# Supplementary figures and images for: Brain-derived exosomal hemoglobin transfer contributes to neuronal mitochondrial homeostasis under hypoxia
Source: eLife. 2025 Jun 23;13:RP99986. doi: 10.7554/eLife.99986 (PMC12185100; doi:10.7554/eLife.99986)

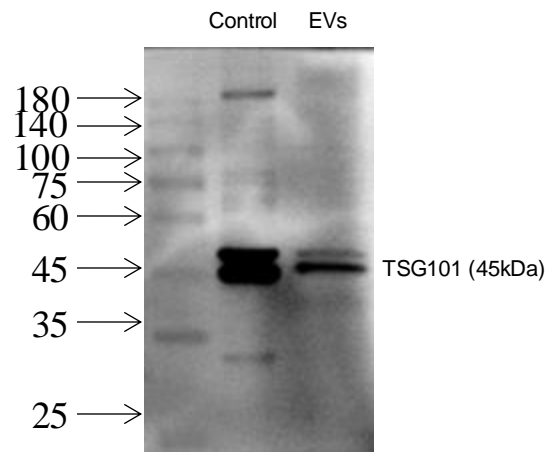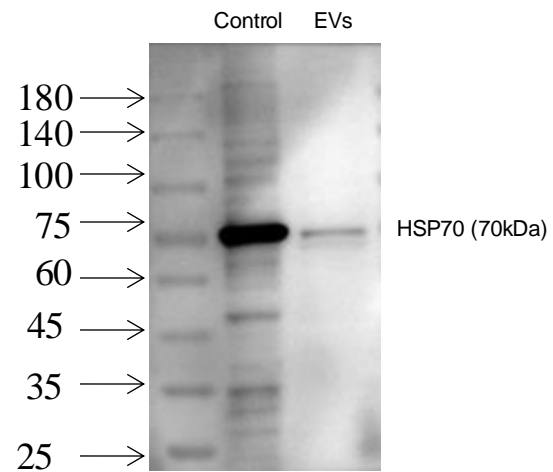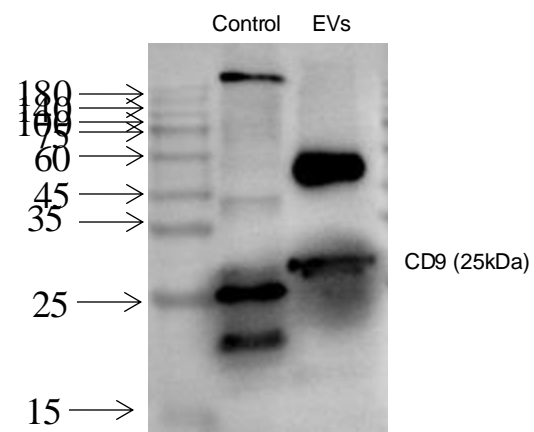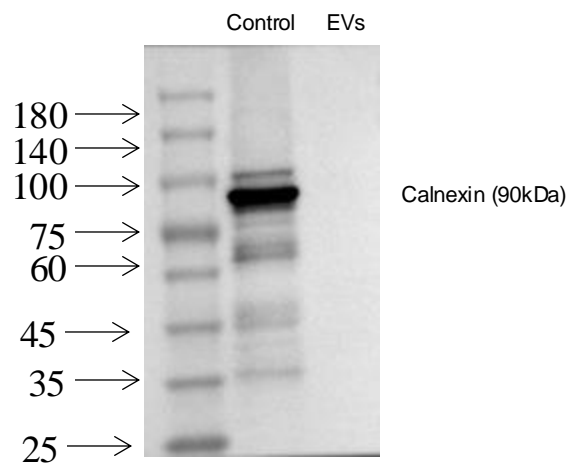

Supplement: Figure 3—source data 1. [file elife-99986-fig3-data1.pdf]

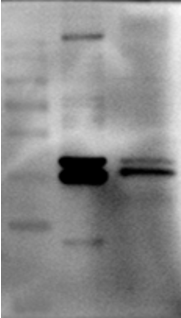

Supplement: Figure 3—source data 2. [file elife-99986-fig3-data2.zip › Figure 3- source data 1/TSG101.png]

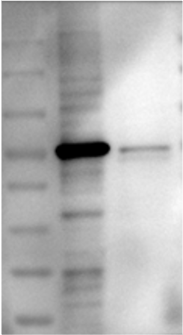

Supplement: Figure 3—source data 2. [file elife-99986-fig3-data2.zip › Figure 3- source data 1/HSP70.png]

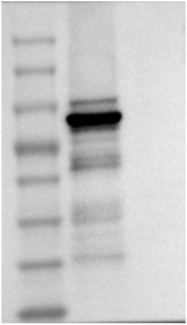

Supplement: Figure 3—source data 2. [file elife-99986-fig3-data2.zip › Figure 3- source data 1/Calnexin.png]

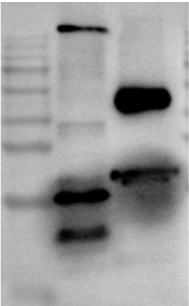

Supplement: Figure 3—source data 2. [file elife-99986-fig3-data2.zip › Figure 3- source data 1/CD9.png]

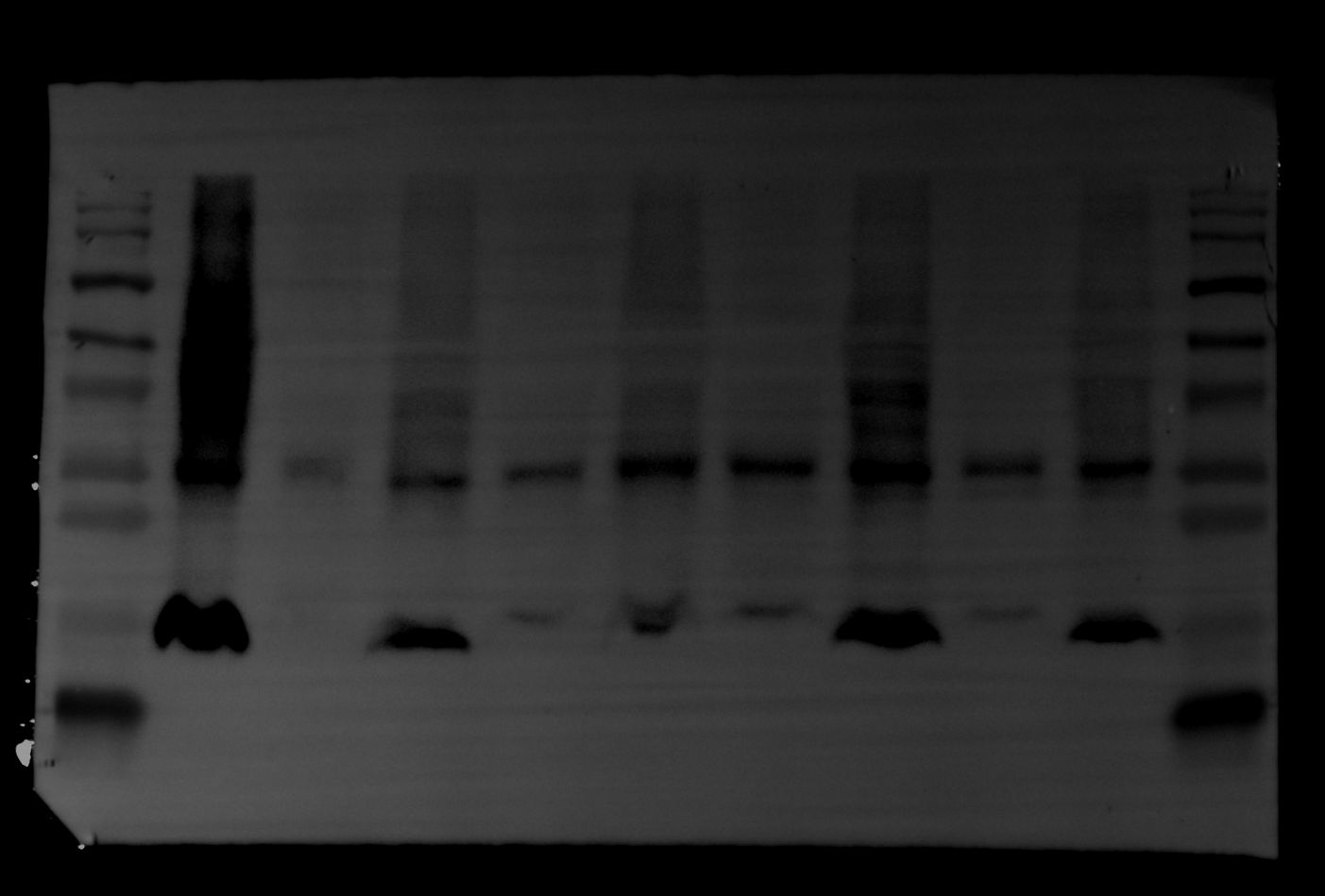

Supplement: Figure 4—source data 2. [file elife-99986-fig4-data2.zip › Figure 4- source data 1/Hba-a1.tif]

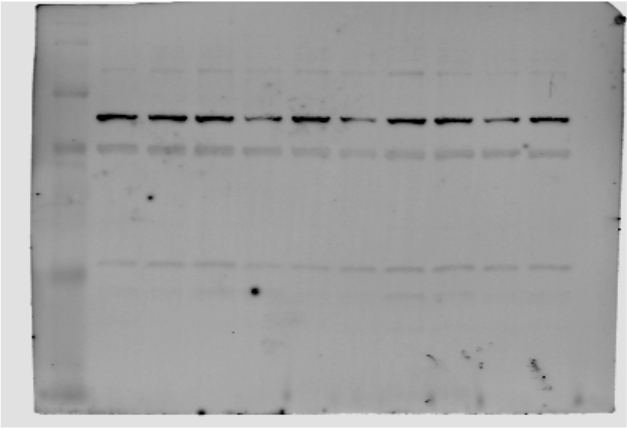

Supplement: Figure 4—source data 2. [file elife-99986-fig4-data2.zip › Figure 4- source data 1/Citrate Synthase.png]

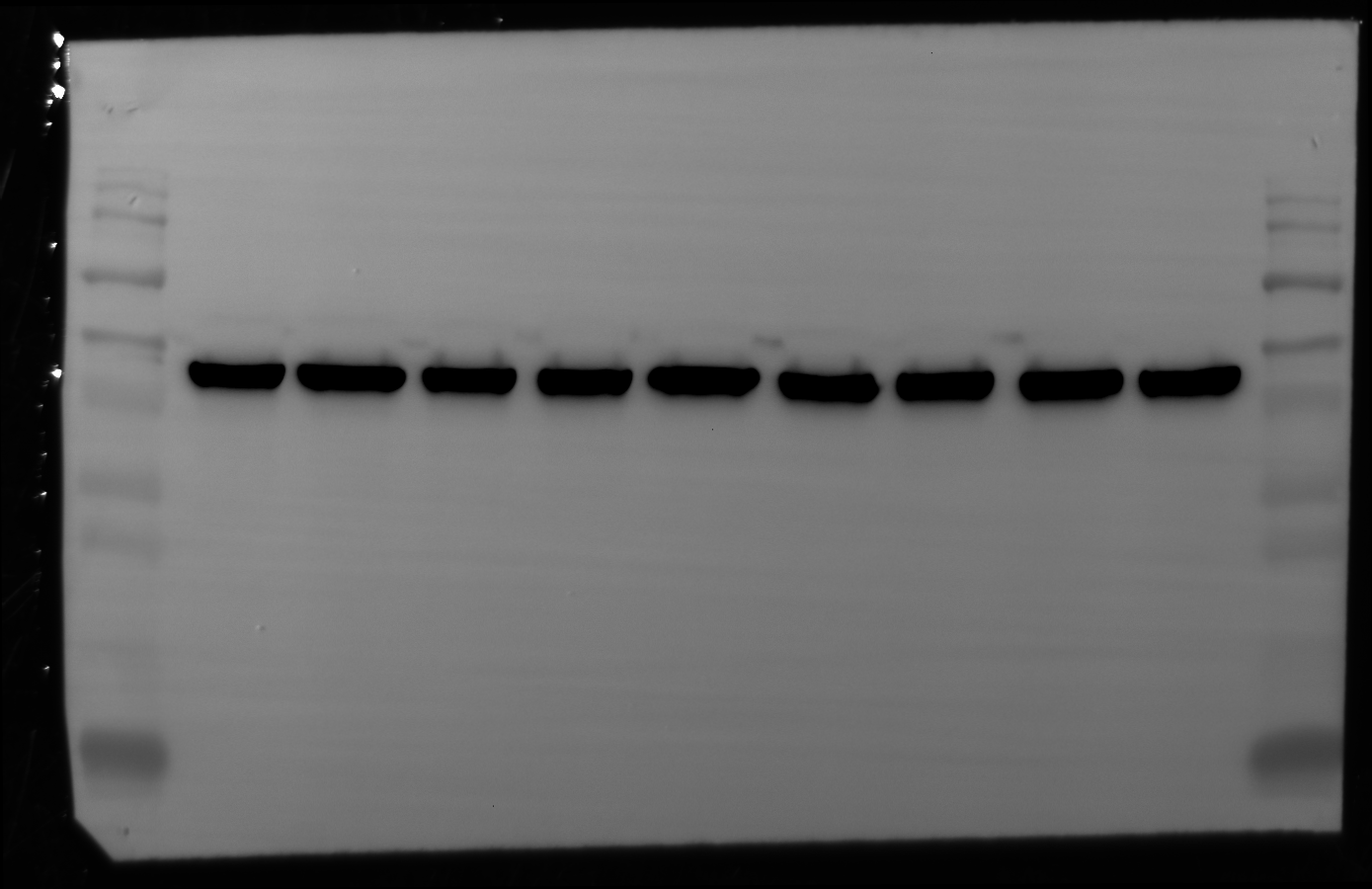

Supplement: Figure 4—source data 2. [file elife-99986-fig4-data2.zip › Figure 4- source data 1/╬▓actin1.tif]

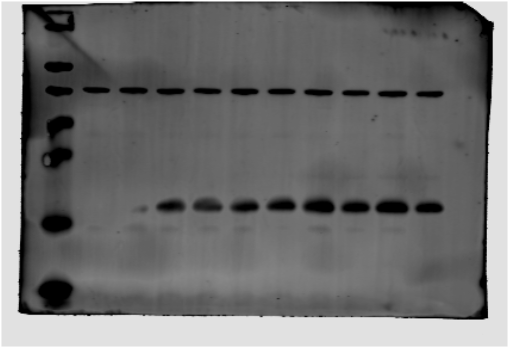

Supplement: Figure 4—source data 2. [file elife-99986-fig4-data2.zip › Figure 4- source data 1/NDUFB8+╬▓actin2.png]

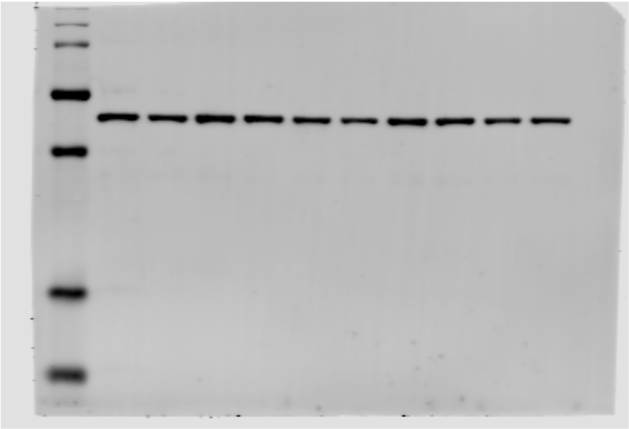

Supplement: Figure 4—source data 2. [file elife-99986-fig4-data2.zip › Figure 4- source data 1/╬▓actin3.png]
