# Supplementary material for: Brain-derived exosomal hemoglobin transfer contributes to neuronal mitochondrial homeostasis under hypoxia
Source: eLife. 2025 Jun 23;13:RP99986. doi: 10.7554/eLife.99986 (PMC12185100; doi:10.7554/eLife.99986)
Supplement: Figure 4—source data 1. [file elife-99986-fig4-data1.pdf]

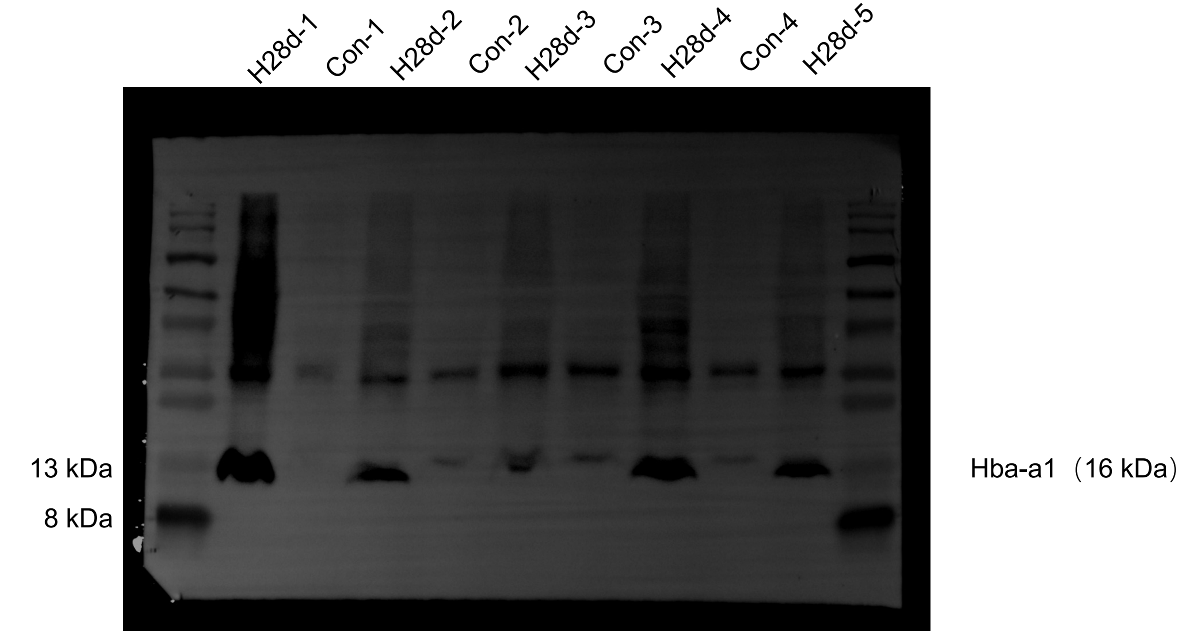
H28d-1
Con-1
H28d-2
Con-2
H28d-3
Con-3
H28d-4
Con-4
H28d-5
13 kDa
8 kDa
Hba-a1（16 kDa）

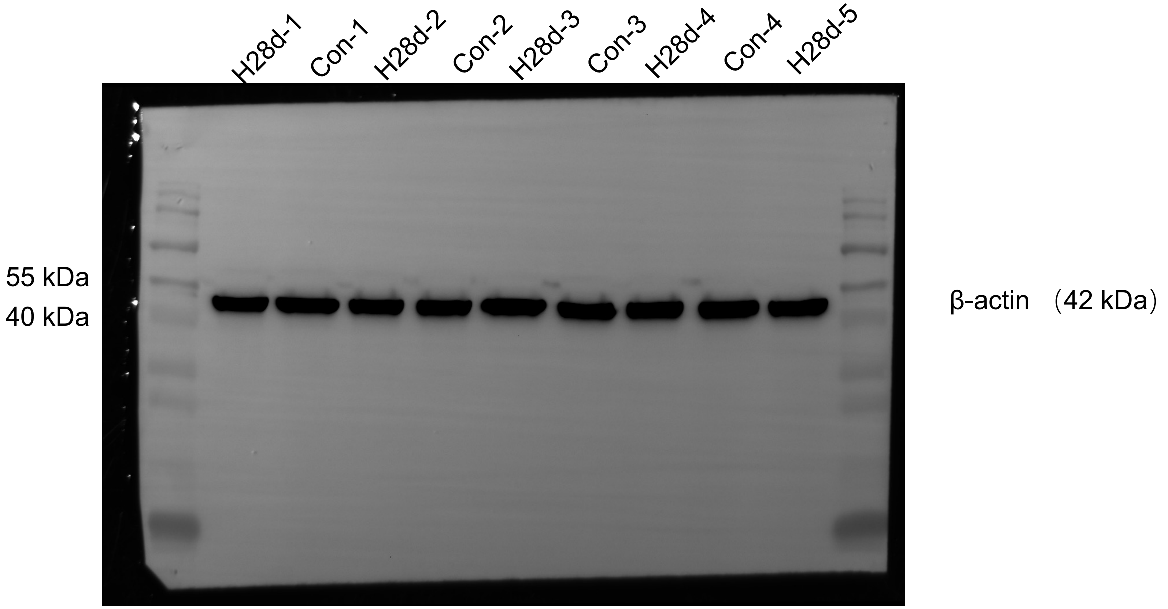
H28d-1
Con-1
H28d-2
Con-2
H28d-3
Con-3
H28d-4
Con-4
H28d-5
55 kDa
40 kDa
β-actin （42 kDa）

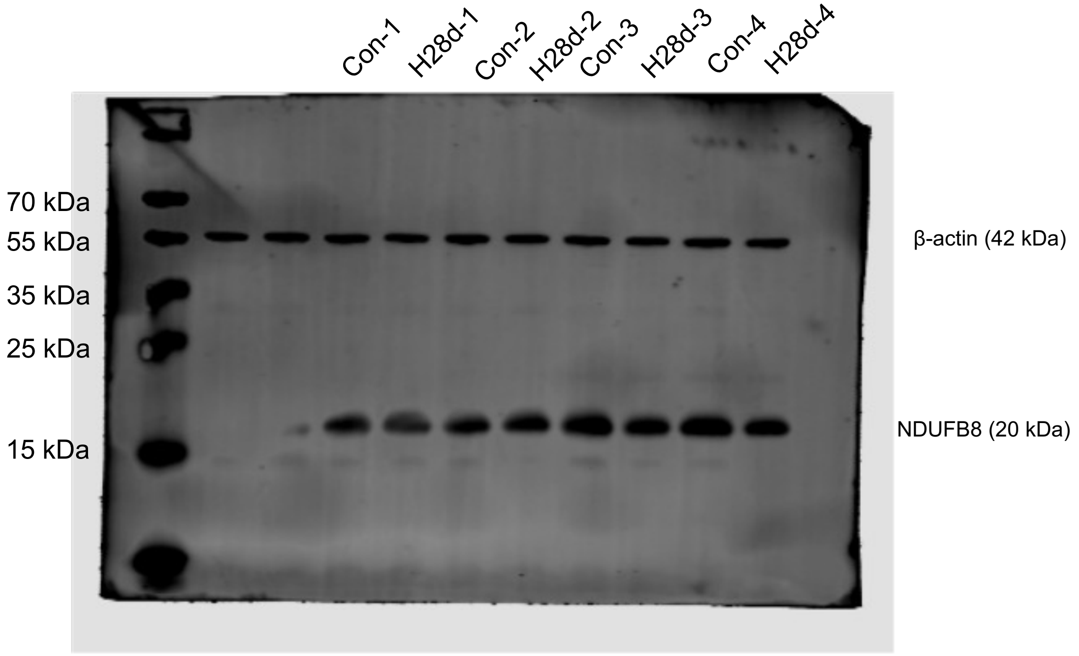
Con-1
H28d-1
Con-2
H28d-2
Con-3
H28d-3
Con-4
H28d-4
70 kDa
55 kDa
35 kDa
25 kDa
15 kDa
β-actin (42 kDa)
NDUFB8 (20 kDa)

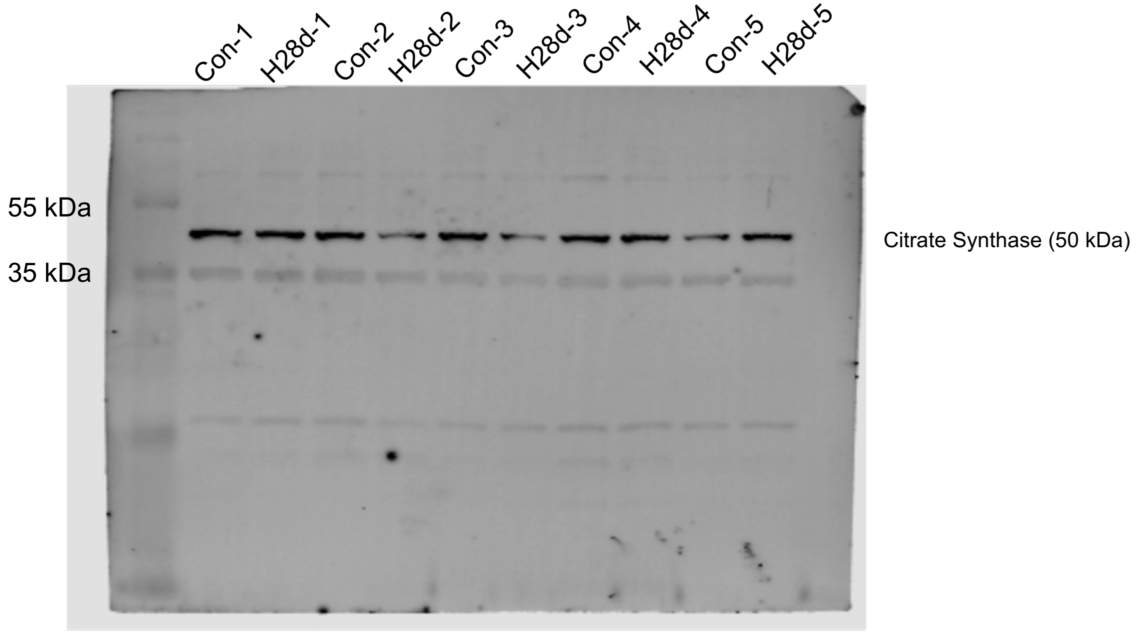
Con-1
H28d-1
Con-2
H28d-2
Con-3
H28d-3
Con-4
H28d-4
Con-5
H28d-5
55 kDa
35 kDa
Citrate Synthase (50 kDa)

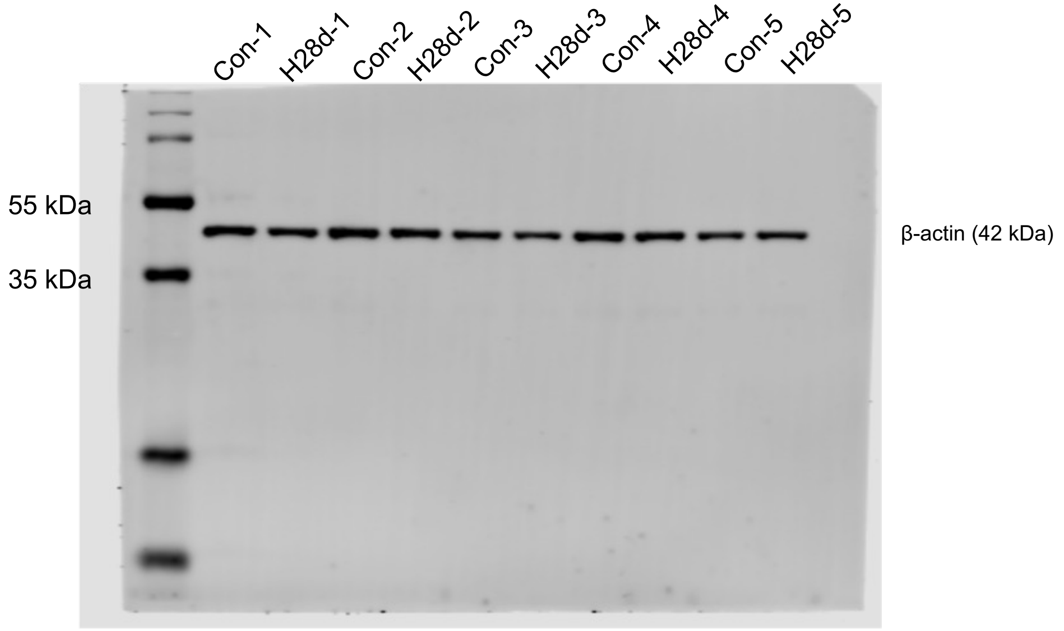
Con-1
H28d-1
Con-2
H28d-2
Con-3
H28d-3
Con-4
H28d-4
Con-5
H28d-5
55 kDa
35 kDa
β-actin (42 kDa)
